# An RNA Aptamer That Specifically Binds to the Glycosylated Hemagglutinin of Avian Influenza Virus and Suppresses Viral Infection in Cells

**DOI:** 10.1371/journal.pone.0097574

**Published:** 2014-05-16

**Authors:** Hyun-Mi Kwon, Kwang Hyun Lee, Byung Woo Han, Mi Ra Han, Dong Ho Kim, Dong-Eun Kim

**Affiliations:** 1 Department of Bioscience and Biotechnology, Konkuk University, Seoul, Republic of Korea; 2 Research Institute of Pharmaceutical Sciences, College of Pharmacy, Seoul National University, Seoul, Republic of Korea; 3 Department of Pediatrics, Korea Cancer Center Hospital, Seoul, Republic of Korea; College of Medicine, Korea University, Korea, Republic Of

## Abstract

The influenza virus surface glycoprotein hemagglutinin (HA) is responsible for viral attachment to sialic acid-containing host cell receptors and it facilitates the initial stage of viral infection. In the present study, we isolated an RNA aptamer specific to the glycosylated receptor-binding domain of the HA protein (gHA1) after 12 cycles of the systematic evolution of ligands by exponential enrichment procedure (SELEX), and we then investigated if the selected aptamer suppresses viral infection in host cells. Nitrocellulose filter binding and enzyme-linked immunosorbent assay (ELISA) experiments revealed that 1 RNA aptamer, HA12-16, bound specifically to the gHA1 protein. Cell viability assay showed that the HA12-16 RNA aptamer suppressed viral infection in host cells by enhancing cell viability. Immunofluorescence microscopic analysis further demonstrated that the HA12-16 RNA aptamer suppresses viral attachment to host cells by neutralizing the receptor-binding site of influenza virus HA. These results indicate that the isolated RNA aptamer can be developed as an antiviral reagent against influenza through appropriate therapeutic formulation.

## Introduction

Avian influenza is an acute viral respiratory disease that causes severe damage to the poultry industry because it results in high mortality. Recent global outbreaks of avian influenza caused by the avian influenza virus (AIV) poses a serious public health threat because of potential transmission among humans. Influenza viruses belong to the family *Orthomyxoviridae* and are further divided into subtypes according to the combination of 2 major immunogenic surface glycoprotein components: hemagglutinin (HA) and neuraminidase (NA), which are present on the surface of viral envelopes [1], [2].

At the initial stage of viral infection, HA binds to the host respiratory cell receptor, which contains sialic acid, allowing the virus to enter the host cell through the endosomal pathway [3], [4]. HA is a homotrimeric transmembrane protein with a globular head and a stem region that are both exposed to the outside of the membrane [5]. These regions contain N-linked oligosaccharides [6], which have been known to affect the functional properties of HA [7], [8]. Glycosylation sites in the peptide sequences are highly conserved, indicating functional significance for HA glycosylation [9]. HA is synthesized as a precursor protein (HA0) that undergoes proteolytic cleavage into HA1 and HA2 subunits; HA1 mediates initial contact with the cell membrane, and HA2 is responsible for membrane fusion [10], [11]. HA is the primary target for antiviral agents such as infectivity-neutralizing antibodies [4] and nucleic acid aptamers [12]. The recombinant HA1 subunit, expressed and purified from bacteria, induces an immune response against the influenza virus in humans [13] and is sufficient for screening antiviral RNA aptamers [14]. The recombinant HA protein, which retains glycosylation, has been expressed and produced in a baculovirus/insect cell system, which exhibited enhanced HA inhibition and virus neutralization [15].

Aptamers are nucleic acid ligands that bind to a specific target molecule with high affinity. They are usually obtained from an oligonucleotide library harboring random sequences by using the SELEX (Systematic Evolution of Ligands by EXponential enrichment) method [16], [17]. Compared with protein antibodies, aptamers have many advantages over protein antibodies, such as high affinity, rapid synthesis, low cost, low-temperature sensitivity, large-scale production, and ease of chemical modification [12]. To date, aptamers have been used in a wide range of applications as research reagents, for medical diagnosis, and as biosensor or therapeutic tools against viruses and cancer. [18], [19]. Previously, our group selected an RNA aptamer against HA1 of subtype H5 AIV, which specifically binds to HA1 and inhibits hemagglutination of erythrocytes *in vitro* [14]. The HA1-specific RNA aptamer HAS15-5 was screened using the recombinant HA1–GST fusion protein that lacks glycosylation because of its expression in bacteria [14]. However, RNA aptamers specific to glycosylated HA rather than unglycosylated HA would be preferable for blocking and inhibiting influenza virus entry into host cells.

In the present study, glycosylated hemagglutinin (gHA1) subtype H5 AIV was expressed from a recombinant baculovirus. gHA1 was expressed in a suspension culture of insect cells by infection with the recombinant baculovirus for glycosylation modifications. We isolated RNA aptamers that specifically bind to the gHA1 protein and demonstrated that the selected RNA aptamer, HA12-16, efficiently inhibited viral infection in host cells and enhanced cell viability.

## Materials and Methods

### Insect cell culture

For suspension culture of insect cells, Sf21 (Invitrogen, Carlsbad, CA) and TriEx Sf9 (Novagen, Darmstadt, Germany) cells were grown in 100 ml of Sf-900 serum-free media (Gibco BRL, Carlsbad, CA) and SFX-Insect cell culture media (Hyclone, Logan, UT), respectively, in 500-ml baffled glass flasks and were incubated at 27°C in a rotary shaker at 90 rpm. For maintaining the insect cells in monolayer cultures, cells were subcultured every 3 days by diluting seed cultures from 5.0×10^5^ cells/ml to a cell density of 2.5×10^4^ cells/ml with fresh media. The cells were then grown in monolayer cultures at 27°C.

### Preparation of recombinant baculovirus

The full-length gene encoding the receptor-binding domain of hemagglutinin (HA1) from influenza virus strain A/wild-duck/Korea/ES/2004 (H5N2) was obtained, as previously described [14]. The *HA1* gene was amplified by PCR and digested with *Xho*I and *Hind*III and then subcloned into the pBAC6 baculovirus transfer vector (Novagen, Darmstadt, Germany), which contained 6 His-tag at the N-terminal and signal peptides for protein secretion in insect cells.

Recombinant pBAC6 plasmids and linearized baculovirus DNA were co-transfected into Sf21 insect cells, as described in the BD BaculoGold baculovirus expression system protocols (BD Biosciences, Franklin Lakes, NJ). Briefly, recombinant pBAC6/HA plasmids (2 µg) and linearized baculovirus DNA (0.5 µg) were mixed in Cellfectin reagent (Invitrogen, Carlsbad, CA) for 5 min and then added to 1 ml of Sf-900 serum-free media. After 15 min of incubation at room temperature, the DNA mixture was added to the Sf21 cells (2.0×10^6^ cells/ ml) in the T25 flask and incubated at 28°C for 4 h while rocking back and forth. Following the rocking incubation, the DNA mixture was removed, and 4 ml of fresh Sf-900 serum-free media was added to insect cells. The insect cells were then incubated at 28°C for 4 days, and the supernatant, which was enriched with recombinant baculovirus (pBAC6/HA), was collected by centrifugation at 1000 × *g* for 5 min.

### Purification of gHA1

The gHA1 protein was expressed in a suspension culture of TriEx-Sf9 cells infected with the recombinant pBAC6/HA baculovirus. TriEx-Sf9 cells that were grown in suspension culture (2.5×10^5^ cells/ml) were infected with the recombinant pBAC6/HA baculovirus with a multiplicity of infection (MOI) of 3.0 and incubated at 28°C for 3 days. Post-infection with the baculovirus, the culture supernatant containing the secreted protein was harvested by centrifugation at 1000 × *g* for 5 min. All viral supernatants were ultra-filtered with the equilibrium buffer (50 mM Tris-HCl, pH 8.0, 100 mM NaCl) through polyethersulfone (PES) membranes of MWCO 5 kDa at a flow rate 120 ml/min using the tangential flow filtration system (PALL, Port Washington, NY) for concentration and diafiltration. The concentrated sample was loaded onto a 5-ml Ni-NTA His Trap affinity column (GE Healthcare, Buckinghamshire, UK), which was pre-equilibrated with the equilibrium buffer. The column was washed twice, and the recombinant gHA1 was eluted with a gradient from 0.1 to 1 M imidazole in the equilibration buffer. The eluted fractions were collected and concentrated with a Centricon Plus-20 (Millipore, Billerica, MA) and were analyzed by 12% SDS-PAGE for the presence of His-tagged gHA1 protein.

The gHA1-containing fractions identified by the band corresponding to 50 kDa were then loaded onto a HiLoad Superdex 200 (GE Healthcare, Buckinghamshire, UK), and eluted at a flow rate 1.5 ml/min. Pure protein fractions were dialyzed against buffer (20 mM Tris-HCl, pH 8.0, 1.0 mM EDTA, 5 mM DTT, 20% glycerol, 0.5% (v/v) Triton X-100, and 150 mM NaCl). Purified gHA1 was quantified using a Bradford protein assay kit (Bio-Rad) using bovine serum albumin (BSA) as the reference standard. The identity of the purified protein was determined by immunoblotting with mouse AIV H5N1 HA polyclonal antibodies (ProSci, Poway, CA) and anti-mouse IgG-horseradish peroxidase conjugate as secondary antibodies (Santa Cruz Biotechnology, Dallas, TX).

### Deglycosylation of the recombinant HA1 glycoprotein

The glycosylation status of the recombinant gHA1 protein was determined with Peptide-*N*-Glycosidase F (PNGase F) that cleaves the complex oligosaccharides at N-linked glycosylations. Briefly, purified gHA1 (3 µg) was denatured in buffer (0.5% SDS and 4 mM DTT), heated at 100°C for 10 min, and subsequently incubated with PNGase F (New England Biolabs, Beverly, MA) according to the manufacturer's protocol. The reaction products were resolved by 12% SDS-PAGE, and the presence of HA1 was subsequently determined by immunoblotting, as described above.

### In vitro selection of RNA aptamers against gHA1

To conduct the SELEX procedure, an RNA pool of random sequences (RNA library) was produced by PCR and by *in vitro* transcription of the DNA template containing 40 random nucleotides, as previously described [20]. The His-tagged HA1 glycoprotein (50 µg) was immobilized on Ni-NTA spin trap columns (GE Healthcare, Buckinghamshire, UK). The random RNA library (6 µg) was denatured at 85°C for 5 min and then incubated at room temperature in binding buffer (20 mM sodium phosphate, pH 7.5, 500 mM NaCl, and 20 mM imidazole). At every iteration of SELEX, negative selection was performed to remove RNAs bound to Ni-NTA resins in columns by passing the RNA pool through separate Ni-NTA spin trap columns. After three iterative rounds of negative selection, the unbound RNAs were collected and reloaded onto new Ni-NTA spin trap columns that were charged with His-tagged gHA1. Next, the column was washed 3 times with binding buffer, and the bound RNA was eluted with elution buffer (20 mM sodium phosphate, pH 7.5, 500 mM NaCl, and 300 mM imidazole). The RNAs were purified by phenol-chloroform extraction and subsequent ethanol precipitation. The purified RNA was subjected to RT-PCR and *in vitro* transcription to generate the RNA library for the next round of SELEX.

The amount of His-tagged HA1 glycoprotein used for SELEX was gradually reduced from 50 to 5 µg to enhance stringency of the selection procedure. After the 12th round of SELEX, the amplified cDNA was cloned into a pGEM T-Easy vector (Promega, Madison, WI) and then transformed into *E. coli* by heat shock at 42°C. The plasmid DNA from each clone was prepared and sequenced, and each RNA aptamer was generated by *in vitro* transcription using the linearized recombinant plasmid that harbored the RNA aptamer sequence. Secondary structures of selected RNA aptamer sequences were predicted by the M-Fold web server, which is based on Zuker's algorithm [21].

### Binding of RNA aptamers to gHA1

To compare the binding affinity of the RNA library at each round, semi-quantitative RT-PCR was carried out as previously described [14]. Briefly, 1.5 µg of gHA1 protein was loaded and immobilized onto the Ni-NTA spin trap column, and 4 µg of RNA pool from each round of SELEX was loaded onto the column. The column containing the RNA-protein complex was then washed 3 times with 200 µl of the binding buffer used in SELEX, and unbound RNAs were collected as flow-through. The RNAs that bound to the gHA1 protein were then eluted from the column by 3 consecutive washes with 200 µl of the elution buffer used in SELEX.

Protein-bound RNAs were then extracted by phenol-chloroform and ethanol precipitation, and the extracted RNAs from each elution were subjected to RT-PCR using aptamer-specific primers. The conditions for PCR were as follows: 11 cycles of 94°C for 30 s, 55°C for 30 s, and 72°C for 30 s. PCR products were electrophoresed on 1.5% agarose gel to measure the amount of DNA that was amplified from each eluant, and the amplified DNA bands on the gel were quantified using the Gel-Pro analyzer software (Media Cybernetics, Bethesda, MD).

Alternatively, to compare each RNA aptamer candidate that originated from the final round of SELEX, a nitrocellulose filter binding assay [22] was performed to measure the binding affinities of RNA aptamers with the gHA1 protein. Each RNA aptamer generated by *in vitro* transcription was dephosphorylated and the 5′-end-labeled with [γ-^32^P] ATP by T4 polynucleotide kinase (New England Biolabs). The 5′-^32^P end-labeled RNA aptamer (5 nM) was incubated with the gHA1 protein (500 nM) in binding buffer (30 mM Tris-HCl, pH 7.5, 150 mM NaCl, and 1.3 mM MgCl_2_) at room temperature for 30 min. The reaction mixture was filtered under vacuum onto double filters composed of a positively charged Hybond N+ membrane (GE Healthcare, Buckinghamshire, UK) beneath the nitrocellulose membranes (GE Healthcare) using a 96-well dot-blot apparatus (Bio-Rad). The membrane was then washed twice with washing buffer (20 mM Tris-HCl, pH 7.5, and 50 mM NaCl) and dried at room temperature.

Filters were then assayed for radioactivity remaining in protein-bound RNAs and free RNAs retained on the nitrocellulose membrane and the hybond N+ membrane, respectively. Radioactivity was measured and quantified on a Cyclone PhosphorImager (Packard Instrument Co., Meriden, CT), and the fraction of protein-bound RNAs was determined using the amount of RNA remaining on the nitrocellulose membrane relative to the total amount of RNA present in both membranes.

### Enzyme-linked immunosorbent assay (ELISA)

Preparation of 3′-biotinylated RNAs occurred by ligation of biotinylated cytidine to the 3′-end of RNA using the 3′-end biotinylation kit (Pierce, Rockford, IL). Briefly, the non-labeled RNAs (50 pmol) were heated at 85°C for 5 min, immediately put on ice, and added to the reaction mixture (40 U RNase inhibitor, 1 nM biotinylated cytidine (bis) phosphate, T4 RNA ligase, and 30% polyethylene glycol) in RNA ligase reaction buffer (50 mM Tris-HCl, pH 7.8, 10 mM MgCl_2_, 10 mM DTT, and 1 mM ATP). After the reaction mixture was incubated at 16°C overnight to increase ligation efficiency, 3′-biotinylated RNAs were then extracted by phenol-chloroform and ethanol precipitation.

For enzyme-linked immunosorbent assay (ELISA), the wells of a nickel-coated plate (Pierce, Rockford, IL) were incubated overnight with the purified gHA1 (0.5 µg) at 4°C. The wells were washed with phosphate-buffered saline with Tween (PBS-T; 0.1% Tween 20 in PBS, pH 7.4) 3 times and blocked with 1% bovine serum albumin (BSA) in PBS at room temperature for 1 h to avoid non-specific binding. After washing, 30 ng of 3′-biotinylated RNAs was added to wells (100 µl in PBS per well) and incubated at room temperature for 2 h. The wells were then washed with PBS-T three times, and the protein-bound biotinylated RNAs were detected by incubation with 100 µl of streptavidin-conjugated horseradish peroxidase (diluted 1∶8000 in PBS-T, Pierce) at room temperature for 1 h. After washing, the color development was initiated by adding 100 µl of 3,3′,5,5′-tetrametylbenzidine (TMB) substrate solution (Pierce, Rockford, IL) to each well, and stopped by adding 2 M H_2_SO_4_. The absorbance of each well was measured at 450 nm using a VICTOR X3 Multilabel Plate Reader (PerkinElmer, Waltham, MA).

### Viability of MDCK cells (MTT Assay)

Madin-Darby Canine Kidney (MDCK) cells (KCLB, Seoul, Korea) were grown in Dulbecco's modified Eagle's medium (DMEM, Hyclone, Logan, UT) supplemented with heat-inactivated 10% fetal bovine serum and 1% penicillin-streptomycin (Hyclone, Logan, UT). Influenza virus strain A/Victoria/210/2009 (Reassortant NYMC X-187) H3N2 grown in the allantoic cavity of 11-day-old embryonated hen eggs was used for infection into MDCK cells. The titer of virus used for infection was evaluated by the infection of MDCK cells with expression, because the tissue culture infective doses lead to 50% infected cells (TCID_50_) [23]. MDCK cells were seeded on 96-well plates (5×10^3^/well) 24 h before the experiment. After 24 h of incubation, the cells were maintained as-is or infected with the influenza virus H3N2 at an MOI of 0.1 TCID_50_ in the presence or absence of the selected RNA aptamer (50 nM). The cells were further incubated in serum-free DMEM at 37°C for 24 h. The cell media were removed after incubation, and cell viability was assayed by adding 5 mg/ml MTT (Sigma) dissolved in PBS to each well and incubation at 37°C for 4 h. The supernatants were aspirated, and the formazan dyes were dissolved in 100 µl/well dimethylsulfoxide (DMSO, Sigma). The absorbance was measured at 570 nm using a VICTOR X3 Multilabel Plate Reader (PerkinElmer, Waltham, MA).

### Immunofluorescence staining analysis of MDCK cells

MDCK cells were placed in 5×10^4^/wells on 8-well chamber glass slides (Nunc, Penfield, NY) prior to the experiment. The cells were washed twice in PBS and then added to a mixture containing the virus and the designated aptamer sample. The MDCK cells were maintained as-is or infected with the influenza virus H3N2 at an MOI of 0.1 TCID_50_ with or without 30 min of pre-incubation with the HA12-16 RNA aptamer (100 nM). MDCK cells were also treated with the RNA aptamer without viral infection as a control. After 24 h of incubation, the cells were fixed with 3% paraformaldehyde followed by permeabilization with 0.5% Triton X-100.

The influenza virus antigen HA was detected by incubating the cultures with a mouse anti-H3 (H3N2) antibody (diluted to 1∶100 in PBS, Abcam, Cambridge, UK). The antibody was incubated at room temperature for 1 h, followed by 3 washes in PBS for at least 5 min per wash. Primary antibodies were detected with FITC-conjugated goat polyclonal anti-mouse immunoglobulin secondary antibodies (Abcam). Nuclei were visualized using DAPI (Sigma) staining. Immunofluorescence images of the cells were obtained using an AxioCam MRc5 digital camera equipped with an Axio Imager A1 microscope (Carl Zeiss, Oberkochen, Germany).

## Results and Discussion

### Purification of gHA1 from insect cells

The HA1 subunit of hemagglutinin in AIV has been previously expressed in and purified from *E. coli* [14], which produces unglycosylated protein. Because HA is an N-glycosylated glycoprotein with a globular head and stem regions [24], proper post-translational glycosylation and protein folding might be required for its function [9]. Hence, we expressed recombinant HA1 in insect cells to obtain the glycosylated HA of AIV by using the baculovirus expression system [15]. The baculovirus expression system can produce post-translationally modified and biologically active recombinant proteins from insect cells. A pBAC6 baculovirus plasmid carrying the full-length *HA1* gene was cloned and transfected into Sf21 insect cells. The morphology of the infected insect cells became larger and irregular (data not shown). Four days post-infection, the secreted recombinant HA1 was collected and purified. The His-tagged gHA1 recombinant protein was purified through the combined use of Ni-NTA His Trap affinity chromatography and gel filtration.

**Figure 1:**
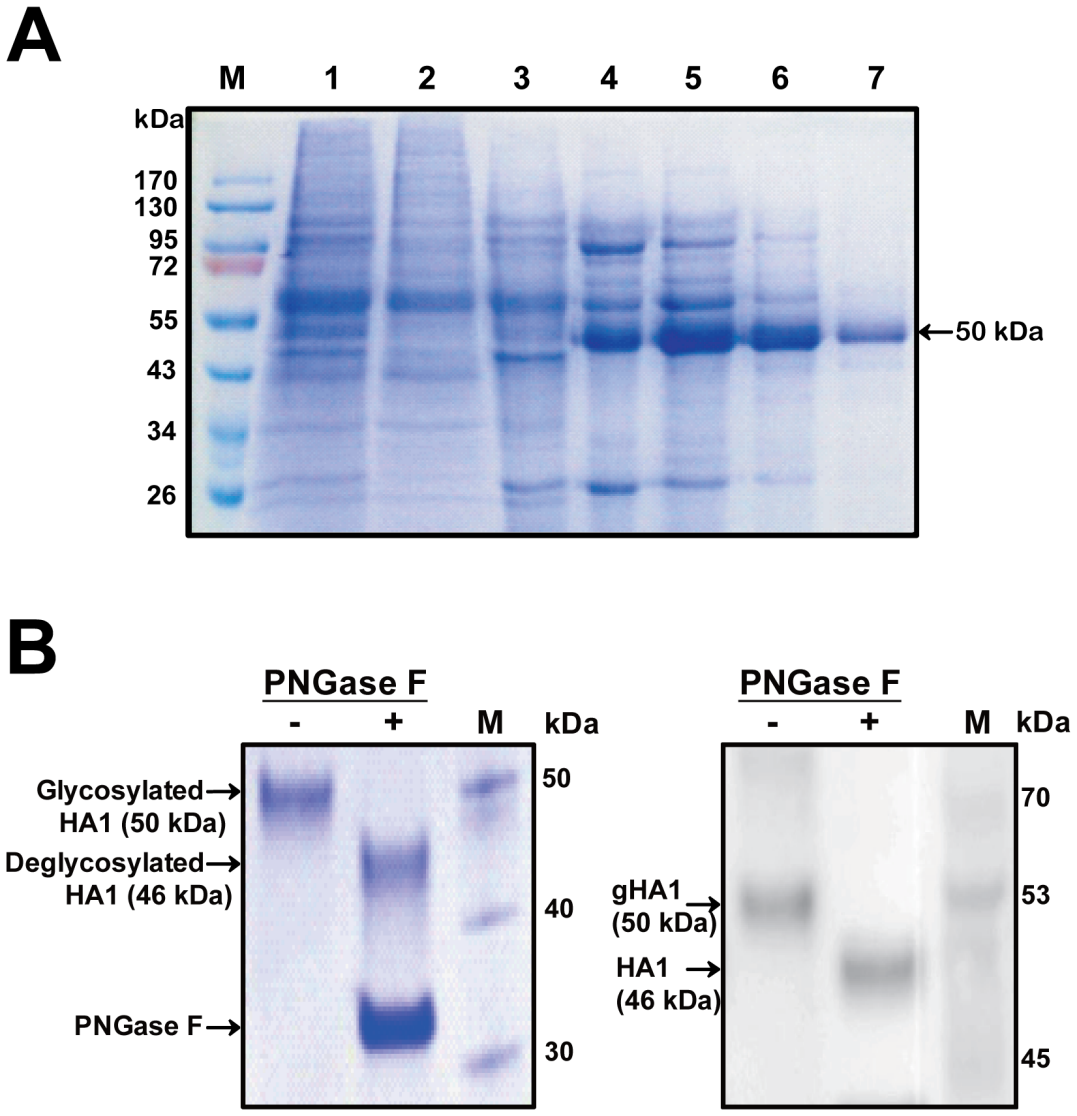
Purification of the gHA1 from insect cells. (A) SDS-PAGE analysis of AIV HA protein expressed in a baculovirus/insect cell system. His-tagged recombinant hemagglutinin protein (gHA1) was purified using Ni-NTA affinity chromatography and gel filtration. Whole supernatant of insect cell (TriEx Sf9) culture was first loaded onto a Ni-NTA affinity chromatography column (Lane 1). Lanes 2 and 3 are the flow-through and washing eluants through the Ni-NTA affinity column, respectively. Lanes 4 to 6 are collected fractions by imidazole elution. Lane 7 is purified gHA1 after gel filtration chromatography (indicated with an arrow). (B) Cleavage of glycans from the purified gHA1. The purified gHA1 (3 µg) was incubated with (+) or without (–) PNGase F, and the reaction mixture was resolved with 12% SDS-PAGE (left panel). HA was probed with anti-HA polyclonal antibody (right panel). The migration of PNGase F treated (+) and untreated (–) recombinant HA1 is indicated with arrows.

The purified protein was separated by SDS-PAGE and identified by immunoblotting analysis. As shown in Fig. 1A, the gHA1 protein fused to His-tag revealed a single band with a molecular weight of 50 kDa in SDS-PAGE. Although the molecular weight of gHA1 is estimated to be about 46 kDa, including 10 kDa of signal sequence plus the His-tag, a slightly higher molecular weight of 50 kDa appeared in SDS-PAGE, probably due to glycosylation.

To confirm whether the purified recombinant HA1 was N-glycosylated during post-translational processing, the purified protein was treated with PNGase F that cleaves glycans from the protein. As shown in Fig. 1B, PNGase F-treated samples had bands that migrated further owing to a loss of glycans, within which the predicted size of unglycosylated HA1 was about 46 kDa. Thus, the purified recombinant HA protein was N-glycosylated and was used for the subsequent RNA aptamer selection procedure (SELEX).

### Selection of RNA aptamers against glycosylated hemagglutinin

**Figure 2:**
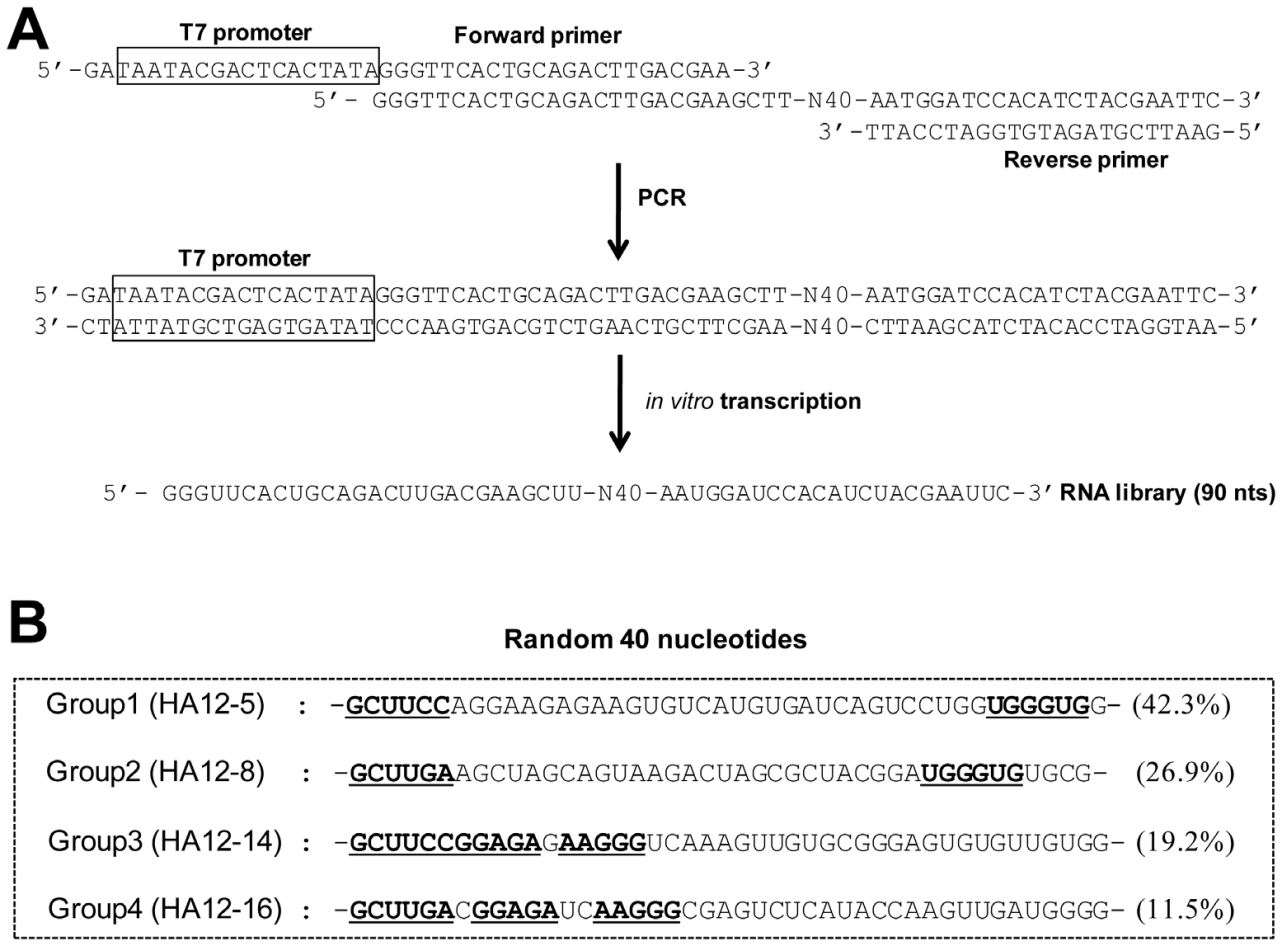
RNA library for SELEX and RNA aptamer sequences. (A) The sequence of the RNA pool for *in vitro* selection is shown. The random RNA library was produced by PCR and *in vitro* transcription of DNA template containing 40 random sequences. (B) The RNAs selected after the 12th round of SELEX were sequenced and grouped based on sequence similarity. Four groups of RNA aptamers were obtained (frequency is depicted in parentheses) and representative RNA sequences of each group are shown (the conserved sequences of each group are underlined).

To obtain antiviral RNA aptamers specific to gHA1 of AIV using SELEX, an RNA library containing 40 random nucleotides (∼10^24^ nucleic acid sequences) was initially prepared by PCR and subsequent *in vitro* transcription of the DNA template (Fig. 2A). Twelve iterative rounds of the selection process were performed by increasing the stringencies of RNA binding to the gHA1 protein as the rounds progressed. Starting from the 5th round, more strict conditions were employed by reducing the protein concentration in each subsequent round: 25 µg (rounds 5–6), 12.5 µg (rounds 7–8), 6.25 µg (rounds 9–10), and 5 µg (rounds 11–12).

After the 12th round of selection and amplification, the amplified cDNA of the final round was subcloned, and 26 individual aptamer clones were sequenced. Based on the degenerated sequences of these clones, sequences of 26 clones were classified into 4 groups (Fig. 2B). The population frequency of the selected RNA aptamers was 42.3>26.9>19.2>11.5% in decreasing percentage ranks (Fig. 2B). Each RNA aptamer group has a conserved sequence that might be considered a binding site specific to the gHA1 protein. The 4 RNA aptamer sequences identified in the 12th selection pool were predicted for the secondary structures of RNA using the M-Fold program, which is based on Zuker's algorithm [21].

**Figure 3:**
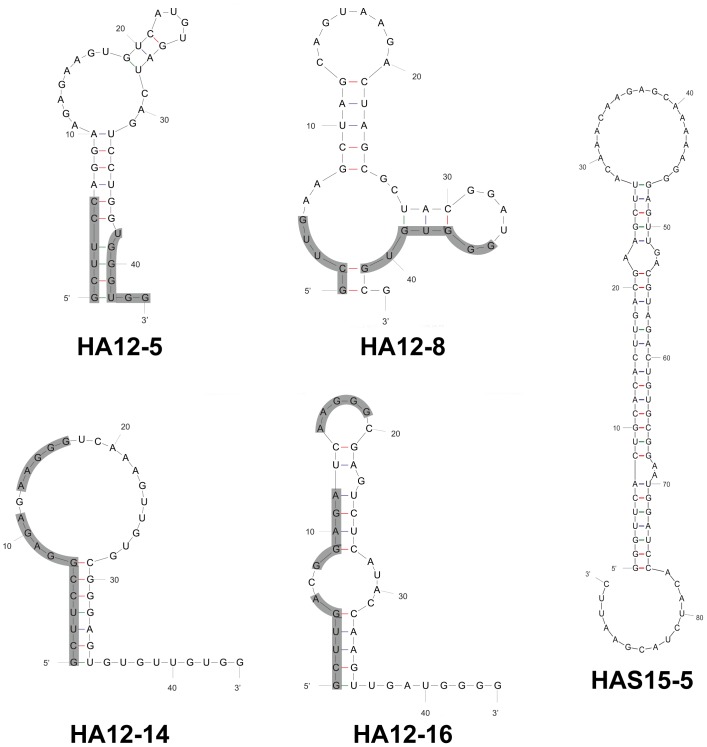
Secondary structure of gHA1 RNA aptamer candidates. The secondary structures of 4 RNA aptamer candidates selected against gHA1 and 1 RNA aptamer (HAS15-5) specific to the unglycosylated HA were predicted by the M-Fold program, based on Zuker's algorithm. The conserved sequences in each RNA aptamer candidate are shown by shadowed lines.

RNA aptamers appear to contain a number of stem and loop structures, in which conserved sequences mostly reside at the stem-and-loop region in the RNA secondary structure (Fig. 3). These conserved sequences in each RNA aptamer are believed to be exposed to facilitate interactions with the HA1 protein [14], [20], [25]. The conserved sequences could constitute a binding motif specific to gHA1, whereas the stem structures of other sequences could merely stabilize the secondary structure of RNA.

**Figure 4:**
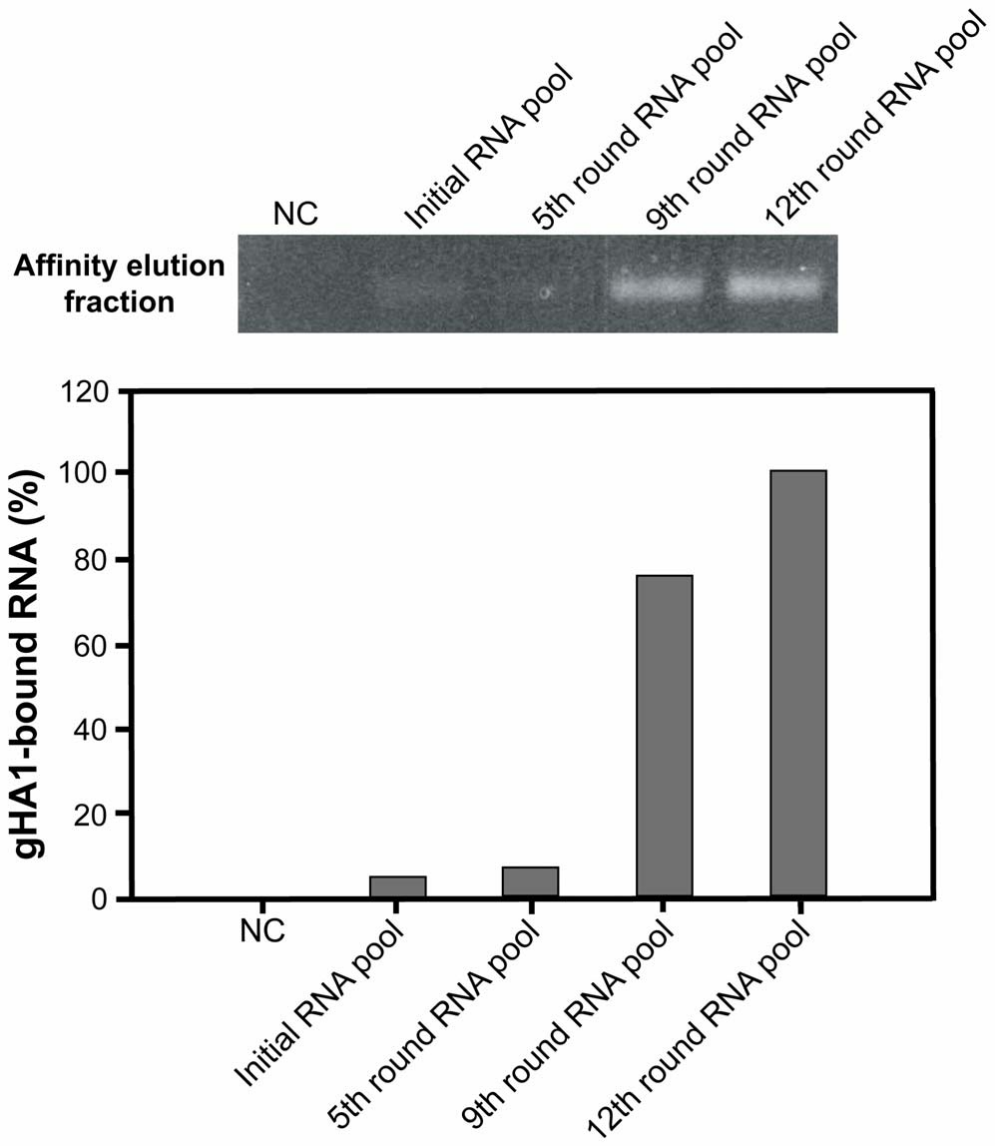
Binding of the RNA SELEX pools to gHA1. The gHA1 protein charged in Ni-NTA spin trap columns was eluted with buffer solution containing the initial RNA pool, the 5th, 9th, and 12th round RNA pools, or no RNA (negative control, NC). Elution with imidazole buffer was collected and subjected to RT-RCR. Amplified cDNAs indicating the gHA1-bound RNAs were stained with ethidium bromide on 1.5% agarose gel after electrophoresis and were quantified. Relative amounts of the gHA1-bound RNAs are shown in the graph.

After 12 rounds of iterative SELEX cycles, the gHA1 protein binding affinity of the 12th RNA pool was compared with that of the initial RNA pool and the 5th and the 9th RNA pools were compared through the semi-quantitative RT-PCR amplification of RNAs bound to the target protein (Fig. 4). For this experiment, the same amount of RNAs from each round was loaded onto an affinity column charged with the gHA1 protein. The affinity-eluted fractions (gHA1-bound RNAs) were collected and subjected to RT-PCR. PCR cycles were limited to 11 cycles to avoid saturation of cDNA amplification [14]. cDNAs were visualized and quantified by ethidium bromide staining on an agarose gel, which reflected the amount of RNA bound to gHA1. Based on quantitation of amplified cDNA, the 12th round RNA pool was more tightly bound to gHA1 than any other round of RNA pool or the negative control containing irrelevant RNA sequences (Fig. 4).

### Binding affinity assay of selected aptamer candidates

**Figure 5:**
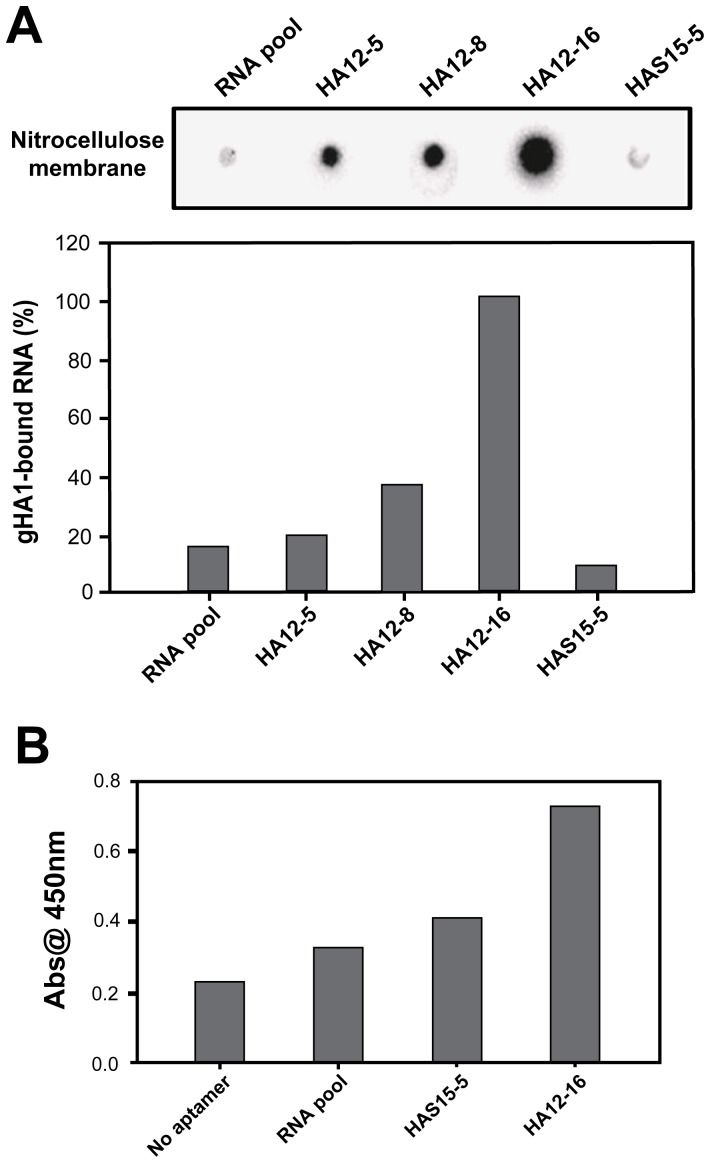
Affinity measurements of selected RNA aptamers to gHA1. (A) Each ^32^P end-labeled RNA aptamer was incubated with gHA1, and the reaction sample was then filtered through a pre-wetted nitrocellulose membrane and applied to the double-filter dot-blot apparatus. The amounts of ^32^P end-labeled RNA aptamers retained on the membrane in the presence of gHA1 were radioactively measured and quantified. Percentage binding to the gHA1 protein relative to the highest value is shown in the graph. (B) The immobilized gHA1 protein (0.5 µg) was incubated with each 3′-biotinylated RNA sample (30 ng) as indicated. After addition of streptavidin-conjugated HRP, the amount of the gHA1-aptamer complex was measured by absorbance reading at 450 nm.

To evaluate the binding affinity of the selected aptamer candidates against gHA1, we performed the double-filter assay for nitrocellulose-filter binding to analyze protein-RNA interactions [22], [26]. The dephosphoylated RNA aptamers were end-labeled with [γ-^32^P] ATP using T4 polynucleotide kinase. Each ^32^P-end labeled RNA aptamer (5 nM) was incubated with gHA1 (500 nM) for 30 min at room temperature, and the reaction mixture was filtered through double filters containing nitrocellulose membrane (0.45 µm). After exposure of the nitrocellulose membrane for visualization of radioactive RNA aptamers bound to the protein, radioactivity was quantified and normalized as shown in Fig. 5A. RNA aptamer HAS15-5, which was previously selected against the unglycosylated HA1 protein [14], did not significantly bind to the glycosylated HA1 protein. In contrast, among the selected RNA aptamer candidates against gHA1, the HA12-16 aptamer displayed the highest affinity.

As an alternative attempt to measure binding affinity of the HA12-16 aptamer to the target protein, gHA1, we carried out a sandwich ELISA assay by adding 3′-biotinylated aptamer RNAs to immobilized target proteins. As shown in Fig. 5B, HA12-16 aptamer RNA exhibited a higher absorbance value compared with other RNAs, such as the initial RNA library and the HAS15-5 aptamer. These results indicate that the selected RNA aptamer (HA12-16) against gHA1 is different from the HAS15-5 RNA aptamer that has been previously selected against the unglycosylated HA1.

### Evaluation of antiviral activity of the selected aptamer

The virus surface glycoprotein HA plays a key role in mediating membrane fusion between the virus and host cellular membranes [4], [12]. We hypothesized that the RNA aptamer specific to gHA1 suppresses viral infection in the host cell by binding, thereby promoting viability in host cells exposed to the influenza virus. To test this hypothesis, we investigated the antiviral effect of the RNA aptamer candidates targeting the gHA1 viral surface protein by performing a host cell viability assay. MDCK cells were infected with the influenza virus H3N2 at an MOI of 0.1 and treated with the selected RNA aptamer candidates. After 24 h of incubation to facilitate viral infection into host cells, cell viability was measured using the MTT reagent.

**Figure 6:**
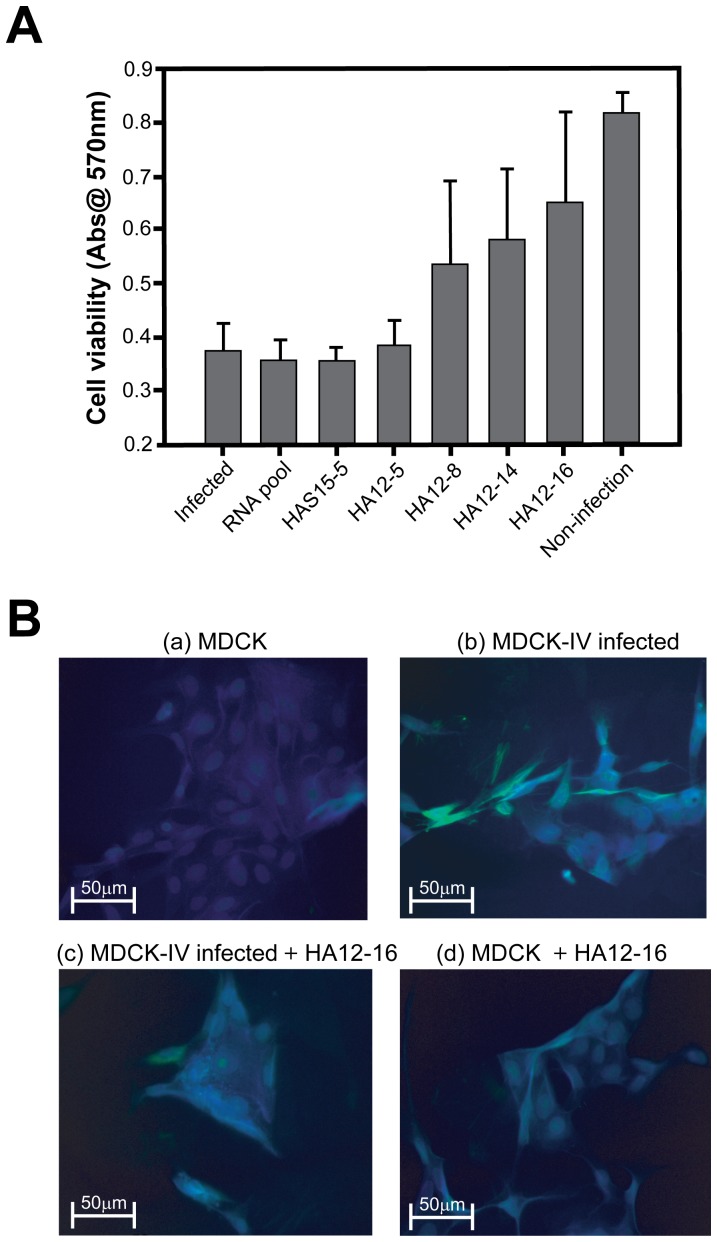
Inhibition of influenza virus infection by the selected RNA aptamer. (A) The antiviral effect of selected aptamers on the viability of MDCK cells infected by the influenza virus was evaluated using the MTT method. MDCK cells were infected with influenza virus H3N2 at an MOI of 0.1 TCID_50_ and then treated with the selected RNA aptamers (5 pmol). The highest protective activity was observed by HA12-16, as compared with other selected RNA aptamers. (B) Microscopic analysis of the effect of HA12-16 on viral infection of cells. Immunofluorescence images of MDCK cells after 24 h of incubation under various conditions were obtained using fluorescence microscopy; (a) MDCK cells only, (b) MDCK cells infected with influenza virus (MOI of 0.1 TCID_50_), (c) MDCK cells treated with the influenza virus and the HA12-16 aptamer (30 pmol), (d) MDCK cells only treated with the HA12-16 aptamer. The cells were fixed with 3% paraformaldehyde followed by permeabilization with 0.5% Triton X-100. The cells were then immunostained to test for the presence of the influenza virus by using mouse anti-HA antibodies and goat anti-mouse polyclonal antibodies conjugated to FITC as secondary antibodies. Nuclei were visualized by staining with DAPI; HA and nuclei are shown in green and blue fluorescence, respectively.

As shown in Fig. 6A, over 50% of the host cells survived in the presence of the RNA aptamers specific to gHA1 except for 1 RNA aptamer candidate (HA12-5). Additionally, the initial RNA pool and RNA aptamer specific to unglycosylated HA1 (HAS15-5) did not inhibit viral infection in host cells compared with the other RNA aptamer candidates. Among the aptamer candidates, the HA12-16 aptamer exhibited the highest antiviral activity by revealing comparable cell viability in the uninfected cells. Of importance, the extent of cell viability is positively correlated with the binding affinity of the RNA aptamer to gHA1, as revealed by the binding assay (Fig. 5A). These results indicate that the most effective aptamer, HA12-16, prevents influenza infection by strongly binding to the gHA1 viral surface protein, thereby decreasing mortality of host cells.

To further validate the antiviral activity of the HA12-16 aptamer in blocking viral binding and entry into host cells, we performed fluorescence microscopy analysis of the cells undergoing viral infection. For the assay, MDCK cells were incubated with the influenza virus in the presence or absence of the HA12-16 aptamer at 37°C for 24 h. The presence of the influenza virus was fluorescently analyzed by FITC-conjugated secondary antibodies bound to anti-HA primary antibodies. MDCK cells infected with the viruses were noticeably fluorescent because of the viruses attached to the cell membrane, whereas addition of the HA12-16 aptamer significantly reduced the FITC-fluorescence owing to the suppression of viral attachment to the cell membrane through aptamer-gHA1 binding (Fig. 6B). When the cells were only treated with the HA12-16 aptamer, FITC fluorescence on the cells appeared to be similar to the one treated with the viruses plus the aptamer. This result suggests that the HA12-16 RNA aptamer suppresses viral attachment to the host cells by neutralizing the receptor-binding site of influenza virus HA, which results in the inhibition of viral replication.

## Conclusions

In this study, we isolated an RNA aptamer specific to the glycosylated receptor-binding domain of an AIV HA protein (gHA1) using SELEX. The *HA1* gene was cloned from subtype H5 of AIV and expressed in insect cells, and the glycosylated recombinant HA1 protein was purified for screening of RNA aptamers. Glycosylation of the purified HA1 protein was confirmed by cleaving glycans with PNGase F. After the 12 rounds of iterative SELEX, the RNA pool that binds to the gHA1 protein was cloned and sequenced, and RNA secondary structures were predicted. Among the 4 representative RNA aptamer candidates, HA12-16 was chosen because of its high binding affinity to gHA1 and suppression of viral infection in host cells. These results suggest that the RNA aptamer can recognize the viral HA, likely at or around the receptor binding region required for the penetration of influenza virus into host cells [27]. Interestingly, the previously selected RNA aptamer against the unglycosylated HA (HAS15-5) failed to inhibit viral infection in host cells. Thus, the HA12-16 aptamer isolated in this study is expected to interrupt influenza invasion through specific binding to the glycosylated ectodomain of HA, which is crucial for viral attachment to the host cell membrane. In future research, the selected RNA aptamers should be modified to improve stability by base modification of nucleotides, capping of RNA fragments, and end-labeling with appropriate chemicals resistant to RNase. If a stable RNA aptamer is combined with an appropriate RNA delivery method, which is necessary for therapeutic use [28], it can be used as an antiviral reagent that is comparable to antibodies and could be used as a therapeutic agent.
